# Quantitative Interpretation of UWB Radar Images for Non-Invasive Tissue Temperature Estimation during Hyperthermia

**DOI:** 10.3390/diagnostics11050818

**Published:** 2021-04-30

**Authors:** Alexandra Prokhorova, Sebastian Ley, Marko Helbig

**Affiliations:** Biosignal Processing Group, Technische Universität Ilmenau, 98693 Ilmenau, Germany; sebastian.ley@tu-ilmenau.de (S.L.); marko.helbig@tu-ilmenau.de (M.H.)

**Keywords:** non-invasive temperature estimation, ultra-wideband, temperature dependent dielectric properties, imaging algorithms, hyperthermia

## Abstract

The knowledge of temperature distribution inside the tissue to be treated is essential for patient safety, workflow and clinical outcomes of thermal therapies. Microwave imaging represents a promising approach for non-invasive tissue temperature monitoring during hyperthermia treatment. In the present paper, a methodology for quantitative non-invasive tissue temperature estimation based on ultra-wideband (UWB) radar imaging in the microwave frequency range is described. The capabilities of the proposed method are demonstrated by experiments with liquid phantoms and three-dimensional (3D) Delay-and-Sum beamforming algorithms. The results of our investigation show that the methodology can be applied for detection and estimation of the temperature induced dielectric properties change.

## 1. Introduction

Hyperthermia has been of interest of many scientific groups for decades. The first paper on hyperthermia was published in 1866 by the German surgeon Carl D. W. Busch (1826–1881) [1]. Hyperthermia is a thermal therapy with relatively low temperature increases by 4–8 °C. It is mostly used as support therapy in cancer treatment, since the thermo effect of hyperthermia can be used to increase radio- and chemo-sensitization of a tumor [2]. In combination with radiotherapy or chemotherapy, hyperthermia can noticeably improve clinical outcomes of cancer therapy [3]. The clinical study of cancer treatment therapies [4] showed that the central part of the tumor is often not responsible for single radiotherapy or chemotherapy but can be reached with applying the hyperthermia treatment. According to the clinical trials, one of the most effective forms of hyperthermia treatment is for tumors in the head and neck region [5].

The main goal of the hyperthermia procedure is to provide a constant required level of heat in the tumor region in order to destroy the cancer cells. But even today, one of the most challenging scientific tasks in this research area is temperature monitoring. Highly accurate temperature control is needed since during hyperthermia procedures, temperature increases should be kept quite low (until 45 °C), in order to prevent damage to healthy cells as well as to monitor the achieved temperature distribution in the tumor to be sure that a sufficient level of heat is applied to all tumor cells. Currently, the most common forms of temperature measurement are minimal-invasive endoluminal thermometry [6] and invasive implementation of fiber optic catheters [7]. Both are painful and only provide limited information on temperature values due to clinical restrictions on the placement of the catheters and their geometry. Since a hyperthermia session duration is around 60 min and sessions should be repeated once a week for a month or more frequently, invasive catheters are not the best option. To improve patient outcomes and patient comfort as well as to reduce the costs of treatment, non-invasive temperature monitoring is of great importance.

Nowadays there are several research activities devoted to non-invasive temperature monitoring including: magnetic resonance imaging (MRI) [8,9], ultrasound based techniques [10,11] and microwave radiometry [12,13] as passive microwave sensing approach. Microwave radiometry is based on the principle of the Planck’s radiation law and measures the thermal electromagnetic radiation emitted by lossy media at temperatures above absolute zero.

Active microwave sensing is a promising alternative which is based on the illumination of the object under investigation with electromagnetic waves in the microwave frequency range and analysis of the transmitted and reflected signals. The detection of the temperature change during the hyperthermia procedure is possible based on the tissue dielectric property changes. The magnitude of decrease or increase of dielectric properties in the microwave frequency range during thermal treatment depends on the tissues water content. High-water content tissues, such as tumor, muscle and liver have larger changes in their dielectric properties during heating in contrast to fat. The low-water content tissues such as fat and bones have significantly smaller temperature dependencies [14]. Changing dielectric properties lead to changing electromagnetic scattering behavior of the illuminated tissue region in the microwave frequency range. Active microwave imaging can be conventionally divided into microwave tomography and ultra-wideband (UWB) radar based techniques.

Microwave tomography as a form of quantitative imaging directly provides estimated information on the dielectric properties of the tissues under tests and is based on the solution for inverse electromagnetic scattering problems [15]. Using a forward model, it solves this inverse problem by iterative minimization of the difference between measured and simulated field values at selected frequencies. Due to the high computational effort of the iterative process, it is time consuming. Already in the 1990s, Chang et al. [16] reported a microwave imaging system which was able to detect temperature caused changes in the electric properties of phantoms. Several research groups continued to develop tomographic methods for non-invasive dielectric properties and accordingly temperature estimations in the human body. In 2014, Haynes et al. [17] presented a microwave imaging system for real-time three-dimensional (3D) imaging of differential temperatures for thermal therapy monitoring accomplished with a precomputed linear inverse scattering solution and Born approximation. But the differential contrast could only be estimated qualitatively. An update to the proposed earlier approach was reported in 2018 [18], where instead of the traditional Born approximation, a non-linear distorted Born iterative method was used to solve the electric volume integral equation and image the temperature changes. The method was verified on numerical phantoms and showed good accuracy reconstruction of the relative permittivity in comparison to previous works. In addition, the research group of the University of Wisconsin performed large-scaled investigations based on anatomically realistic numerical phantoms and developed several reconstruction techniques [19,20]. 

A good alternative for microwave tomography is microwave temperature monitoring based on the UWB radar technology. The main advantage of the radar based microwave imaging is wide frequency band measured data for dielectric properties analysis. Moreover, the UWB approach does not require the iterative solution of inverse scattering problem and accordingly has lower computational effort. UWB radar based imaging algorithms detect and localize strong scatterers by means of coherent summation of the reflected signal energy and are widely used in early stage microwave breast tumor detection [21,22,23,24]. This qualitative method images temperature induced varying tissue reflectivity due to its changing dielectric properties, which has to be converted into temperature values. Even small changes of relative permittivity (ε) and effective conductivity (σ) can be detected by ultra-wideband technology [25]. 

The purpose of this contribution is to present the results of our investigation towards quantitative exploitation of UWB radar images for non-invasive tissue temperature estimation. The paper is organized as follows. In the Section 2.1, the methodology is described and illustrated by a flowchart. Section 2.2 is devoted to the microwave imaging algorithms, especially beamforming and its adapted version. The estimation algorithm based on the temperature dependent change of the reflection coefficient at the boundary between background material and target is described in Section 2.3. Section 2.4. shows the relation between dielectric properties and temperature values. Section 2.5 is dedicated to the measurement setup and measurements description. The phantom materials are specified, and their dielectric properties measured by means of dielectric spectroscopy are introduced. Before imaging of temperature changes can come into focus, the detectability by means of UWB radar technology is demonstrated and its limitations are investigated by experiments simulating the tumor heating during hyperthermia treatment in Section 3.1. One of the remaining challenges is the proper quantitative translation of these differential signal amplitudes into temperature values. Section 3.2 shows the quantitative experimental results, the distribution of the estimated dielectric properties in the 3D target and their comparison to the measured ones. Finally, the achieved results, limitations of the method and further enhancements of the approach are discussed. The results of this investigation allow us to get closer to the development of electromagnetic devices for temperature monitoring during the hyperthermia treatment.

## 2. Materials and Methods

### 2.1. Methodology

**Figure 1:**
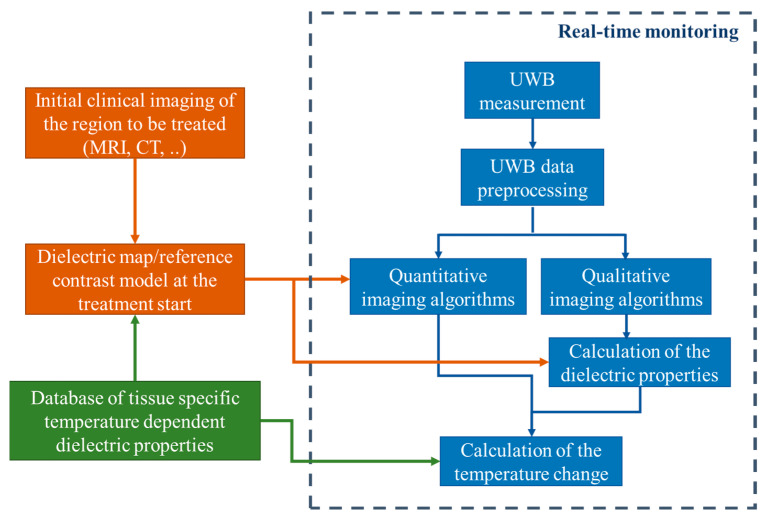
Flowchart of the tissue temperature monitoring methodology.

Generally, our methodology of non-invasive quantitative temperature monitoring for hyperthermia is based on continuous estimation of the changing tissue dielectric properties in the area of interest during the treatment by means of microwave sensing. The flowchart of this approach is presented in Figure 1.

Firstly, the knowledge of the temperature dependency of relative permittivity and effective conductivity of specific tissues in the body region to be treated by hyperthermia in the relevant microwave frequency range is a prerequisite for transforming the ongoing dielectric values into current temperatures. Although it must be stated that this part still needs additional investigations, several temperature dependent studies of animal as well as human tissues are already available in the literature [26,27,28]. Recently, an extensive UWB temperature dependent dielectric spectroscopy study of porcine tissue and blood in the frequency range from 0.5 GHz to 7 GHz and in the temperature range between 30 °C and 50 °C was published by the authors as well [14].

The temperature dependent dielectric properties are described by the complex relative permittivity (*ε*) and the effective conductivity (*σ*):

(1) $$\begin{array}{c} \underline{\varepsilon }(\omega ,\vartheta )=\varepsilon^{\prime }(\omega ,\vartheta )-i\varepsilon^{\prime \prime }(\omega ,\vartheta )\\ \sigma (\omega ,\vartheta )=\omega \varepsilon_{0}\varepsilon^{\prime \prime }(\omega ,\vartheta ) \end{array}$$

where the real part $\varepsilon^{\prime }$ represents the relative permittivity, the imaginary part $\varepsilon^{\prime \prime }$ the relative dielectric loss, $\varepsilon_{0}$ the permittivity of free space, ω=2πf the angular frequency and ϑ the temperature. The database of tissue specific temperature dependent dielectric properties is required to calculate the change of temperature in the area of treatment based on the values of the relative permittivity and effective conductivity of the heated tissues obtained from imaging.

The next step in our approach is patient individual preparation of dielectric maps and mathematically defined or simulated contrast models of the region of interest as input (initial guess) for microwave imaging. Considering that hyperthermia is used as supporting therapy for cancer treatment, the availability of a computer tomography or MRI scan of the patient can be assumed. Based on an initial body core temperature measurement of the patient and the mentioned database, an initial guess of the dielectric properties of the specific tissues to be treated can be defined at the start of the treatment.

The third part is the non-invasive tissue temperature monitoring by means of UWB sensing. The UWB measurement is ongoing during the whole hyperthermia procedure. In practice it will likely be the case that the heating phase and temperature measurement phase alternate in order to protect the sensitive sensor hardware from the high-energy radiation produced by the hyperthermia applicators during heating. The spatial reconstruction of changing dielectric properties inside the human body is made via imaging algorithms. Depending on the type of algorithm, the dielectric properties are calculated directly (quantitative imaging) or in an intermediate step using the reconstructed contrast function or reconstructed reflection intensity (qualitative imaging). Afterwards, the change of the temperature in the area of interest is estimated based on the knowledge of temperature dependency of the specific tissues. The detailed description of this part is presented in the following Section 2.2, Section 2.3, Section 2.4 and Section 2.5.

### 2.2. Imaging Algorithms

The methodology for tissue temperature monitoring described above should not be limited to a special imaging algorithm. It is important to investigate which algorithms better meet the specific requirements for this application (e.g., resolution, robustness, suitability for real-time analysis, computational effort, etc.).

Both types of algorithms need initial information. Microwave tomography starts with a spatial distribution of initial dielectric properties in the region to be imaged. UWB radar approaches assume an averaged propagation velocity. On the one hand, avoiding iterative solutions of inverse problems, the computational effort of UWB radar approaches is much less than of microwave tomography. On the other hand, qualitative imaging approaches do not directly provide absolute values of relative permittivity and effective conductivity, which has to be done afterwards. 

**Figure 2:**
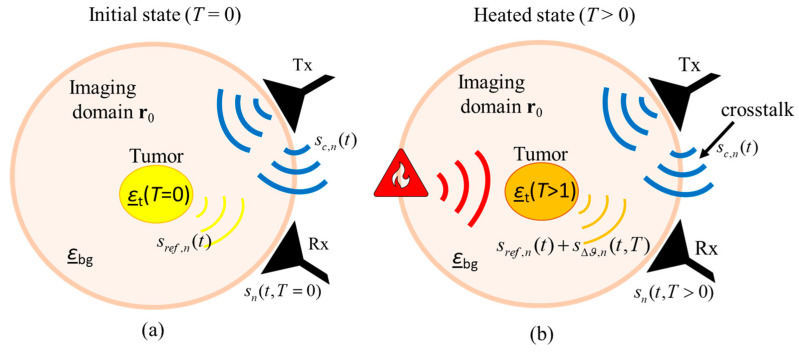
Schematic of the measurement scenario of temperature estimation during hyperthermia by means of UWB sensing (**a**) before start of treatment and (**b**) during treatment.

The received UWB measurement signal of channel *n* consists of two components:

(2) $$s_{n}(t,T)=s_{c,n}(t)+s_{h,n}(t,T)$$

where *t* is the signal propagation time, *T* is the treatment time, $s_{c,n}(t)$ is the static clutter including channel specific antenna crosstalk as well as static signal components caused by reflections inside the measurement radar system and $s_{h,n}(t,T)$ is the ongoing and patient specific signal determined by the tissue region to be monitored during the hyperthermia treatment. After the crosstalk removal, the monitoring relevant signal part remains:

(3) $$s_{h,n}(t,T)=s_{ref,n}(t)+s_{\Delta \vartheta ,n}(t,T)$$

where $s_{ref,n}(t)=s_{h,n}(t,T=0)$ is the reference signal representing the tissue region to be treated at beginning of hyperthermia session and $s_{\Delta \vartheta ,n}(t,T)$ is the signal representing the change of dielectric properties with ongoing treatment time *T* due to changing tissue temperature. Figure 2 illustrates the measurement scenario, including the related signal components.

In this paper, the focus is on UWB radar imaging based on Delay-and-Sum (DAS) algorithm [21,24]. DAS is a qualitative beamforming imaging algorithm developed for time domain, which is based on the principle of coherent addition of backscattered radar signals after clutter removal, which are collected while illuminating the target. The main advantages of the DAS algorithm are its simplicity, robustness and short computation time. 

The DAS beamformer equation can be written as:

(4) $$I\left(r_{0},T\right)=\sum_{n=1}^{N}s_{h,n}\left(t_{0}+\tau_{n}(r_{0}),T\right)$$

where *N* is the number of channels (pairs of Tx and Rx antennas), *t_0_* is the time where the electromagnetic wave takes off the transmitter antenna and *τ_n_* is the channel dependent time of flight–distance from the transmitting antenna to the focal point $r_{0}=(x,y,z)$ and back to the receiving antenna. It is calculated based on the assumed mean propagation speed. The signals received at each channel are time-aligned for each focal point $r_{0}$ and coherently summed. The summed signal is assigned to the reflection intensity of the focal point and this process is repeated for all focal points within the region of interest. 

Measurement systems with antenna movement allow extending the number of included signals. For our case, the whole antenna array can be rotated around the phantom, which yields.

(5) $$I\left(r_{0},T\right)=\sum_{n=1}^{N}\sum_{m=1}^{M}s_{h,n}{}_{{}_{m}}\left(t_{0}+\tau_{n_{m}}(r_{0}),T\right)$$

where *M* is the number of different rotation angles. 

In order to improve the quality of the reconstruction, we investigated different adapted versions of summation. For the present paper, we applied Equation (6) which avoids a reduced target representation in the image due to mutual extinction of channels with different sign by averaging the absolute image values of each channel. On the other hand, the expected value of image regions without scatterers (i.e., averaging noise) is not zero and will only be reduced with an increasing *M*.

(6) $$I\left(r_{0},T\right)=\sum_{n=1}^{N}\left|\sum_{m=1}^{M}s_{h,n_{m}}\left(t_{0}+\tau_{n_{m}}(r_{0}),T\right)\right|=\sum_{n=1}^{N}\left|I_{n}\left(r_{0},T\right)\right|$$

### 2.3. Permittivity Estimation 

Because qualitative radar imaging does not directly provide dielectric property values, the next step of our approach is the estimation of relative permittivity and effective conductivity in the region of interest from the reconstructed UWB radar images. We assume that the image values $I(r_{0},T)$ in the region of treatment are depending on two parts: the dielectric contrast between background material and tumor tissue, as well as on the aggregation of all other temperature independent influencing parameters (e.g., the radar cross section of the tumor, signal path attenuation, impulse response of radar and antennas, etc.) which we do not know exactly, and we cannot quantify separately, especially because the treatment area is located in the near field. Therefore, they are summarized within the parameter *F*:

(7) $$I(r_{0},T)=\underline{F}(r_{0})\cdot \underline{\Gamma }(r_{0},\omega ,T).$$

Since all parameters included in *F* remain constant during hyperthermia treatment, only the dielectric contrast between background and tumor will change [29]. For the sake of simplicity, specular reflection is assumed at the boundary between the background medium and the tumor and approximate the effect of dielectric contrast in (Equation (7)) by means of the complex reflection coefficient Γ:

(8) $$\underline{\Gamma }(r_{0},\omega ,T)=\frac{\sqrt{{\underline{\varepsilon }}_{bg}(r_{0},\omega )}-\sqrt{{\underline{\varepsilon }}_{t}(r_{0},\omega ,T)}}{\sqrt{{\underline{\varepsilon }}_{bg}(r_{0},\omega )}+\sqrt{{\underline{\varepsilon }}_{t}(r_{0},\omega ,T)}}$$

where *ε*_bg_ and *ε*_t_ are the complex relative permittivities of the background and tumor region to be heated, respectively. 

From the initial clinical imaging and the database of tissue specific dielectric properties (see Figure 1), we know the correct localization and size of the tumor and can specify the permittivity of tumor and background at the beginning of treatment. The reflection coefficient and the corresponding UWB image at the starting time *T* = 0 are used as reference and refer to ${\underline{\Gamma }}_{ref}(r_{0},\omega )=\underline{\Gamma }(r_{0},\omega ,T=0)$ and $I_{ref}(r_{0})=I(r_{0},T=0)$, respectively. During the treatment, we relate the ongoing images to this reference image:

(9) $$\frac{I(r_{0},T)}{I_{ref}(r_{0})}=\frac{\underline{\Gamma }(r_{0},\omega ,T)}{{\underline{\Gamma }}_{ref}(r_{0},\omega )}.$$

In this way, the effect of $\underline{F}(r_{0})$ is eliminated, and the changing permittivity ${\underline{\hat{\varepsilon }}}_{t}(r_{0},\omega ,T)$ can be estimated:

(10) $${\underline{\hat{\varepsilon }}}_{t}(r_{0},\omega ,T)={\underline{\varepsilon }}_{bg}(\omega ){\left(\frac{I_{ref}(r_{0})-{\underline{\Gamma }}_{ref}(r_{0},\omega )I(r_{0},T)}{I_{ref}(r_{0},)+{\underline{\Gamma }}_{ref}(r_{0},\omega )I(r_{0},T)}\right)}^{2}.$$

It has to be emphasized that this approach is only able to estimate the permittivity and temperature, respectively, inside the target to be treated. This is because outside this area ${\underline{\Gamma }}_{ref}(r_{0},\omega )=0$, Equation (10) yields ${\underline{\hat{\varepsilon }}}_{t}(r_{0},\omega ,T)={\underline{\varepsilon }}_{bg}(\omega )$ independently from $I(r_{0},T)$.

### 2.4. Temperature Calculation

Based on the database of tissue specific temperature dependent dielectric properties (see Figure 1) and the estimations described in the previous section, the temperature can finally be calculated. This section gives a short overview of expectable permittivity changes during hyperthermia.

The study of Lazebnik et al. [26] shows a decrease of permittivity for liver tissue at 2.45 GHz equal to 0.1 per degree Celsius. Another study [30] demonstrates temperature and frequency dependencies of dielectric properties of porcine fat, muscle, liver and tumor phantom in a microwave frequency range. At the frequency of 3 GHz, the decrease of permittivity per 1 °C for tumor mimicking material is equal to approximately 0.25, 0.1 for muscle and liver and 0.03 for fat tissue. In the work of Faktorova [27], temperature dependencies at the frequency 10 GHz of porcine bone marrow and muscle tissues are presented. While dielectric properties of bone marrow do not show temperature dependency, the increase of permittivity of muscle sample is equivalent to 0.1 per 1 °C. 

Based on the measurements presented in our previous work [14], the decrease of relative permittivity of porcine liver and muscle at 2 GHz is equal to 0.1 per 1 °C of the temperature increase. Fat tissue shows a much smaller temperature dependency of about 0.01 per 1 °C. These coefficients correlate well with the experimental studies mentioned above, so that these relations can be used to convert dielectric property values to temperatures. In practical clinical scenarios during hyperthermia therapy, the increase of temperature should be in the range from 4 to 8 °C to provide quality treatment. Thus, due to the relations mentioned above, the expected decrease of relative permittivity will be about 0.4–0.8 for tumorous and high-water content tissues and approximately 0.04–0.08 for low-water content tissues such as fat.

### 2.5. Measurement Setup and Measurements

In this paper, we evaluate the approach based on M-sequence radar technology developed at Technische Universität Ilmenau, Germany. M-sequence UWB radar technology uses pseudo-noise stimulation signals (M-sequences) generated by high-speed digital shift registers. The signal energy is distributed equally over time; thus, the signal magnitudes remain low. The reduced voltage exposure of the medium under test makes this technology suitable for medical applications. The measurement signal of M-sequence radar technology is the impulse response of the scenario under test and is created by means of cross correlation between received signal and ideal M-sequence [31]. Therefore, the amplitude unit is arbitrary. 

The multiple input multiple output (MIMO) radar device applied in this work is a baseband system (bandwidth 6.5 GHz) including 8 transmitters and 16 receivers, which results in 128 channels. For this process, 24 passive bow-tie dipole antennas are placed in quasi direct contact with the tissue phantom arranged in 8 groups of 3 antennas, with each group displaced by 45° around the phantom and at different heights. The bow-tie antennas, implemented on FR4 printed circuit board substrate (0.8 mm) with dimensions of 10 mm by 5.6 mm, are differentially fed by passive baluns. A rotational scanner is used to rotate the antenna array around the phantom and increase the amount of measurement channels.

**Figure 3:**
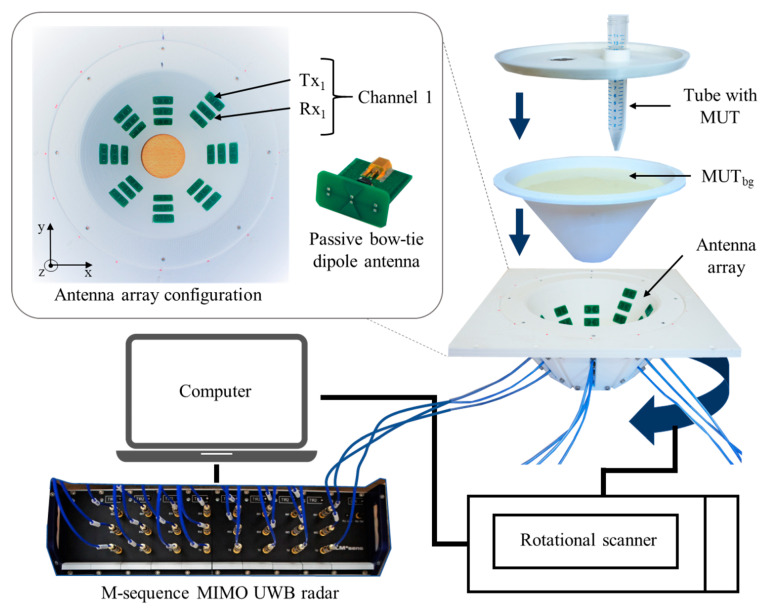
Measurement setup for preliminary investigations towards tissue temperature estimation. MIMO stands for multiple input multiple output, UWB for ultra-wideband and MUT for material under test, index in MUT_bg_ stands for background.

The scheme of the measurement setup is presented in Figure 3. It includes a 3D printed polylactic acid cup filled with the background material and a plastic lab tube (Ø 1.5 cm, volume 12 mL) filled with tumor mimicking the material under test (MUT). The cup has a shape of truncated cone with an upper diameter of 15 cm, lower diameter of 5 cm and height of 8.5 cm. In the presented measurements, we mimic temperature changes in the heated tumor by directly changing the permittivity of the tumor tissue mimicking material. The use of liquid tumor imitates in the test tube provides the opportunity for fast replacements and accurate manipulations without excessive interaction with the measurement setup. The tube is inserted at position x = 2.3 cm, y = 0 cm.

The cup is filled with cream as background medium of known dielectric properties. Due to its composition (emulsion of fat and water), cream is well suited to mimic the dielectric properties of average neck tissue [32,33]. Additionally, the liquid physical state of cream is appropriate for the current phantom configuration.

**Figure 4:**
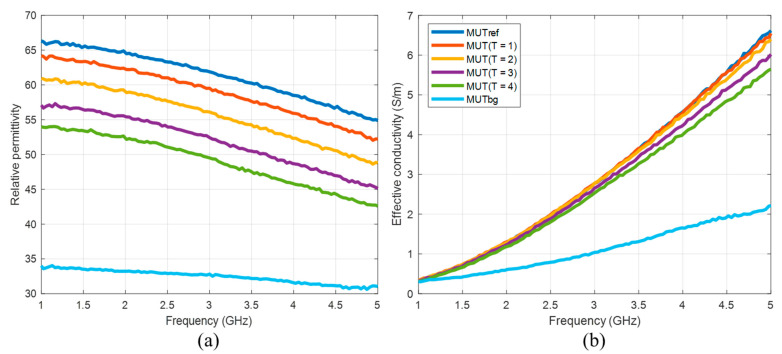
(**a**) Relative permittivity and (**b**) effective conductivity of MUTs as a function of frequency.

For tumor imitation, we use mixtures of distilled water and pure acetone (99.8%). Acetone can be chosen due to its values of dielectric properties in the microwave frequency range and their low dispersity. The mixture of 30 vol% acetone and 70 vol% distilled water mimics the tumor to be treated before start of heating. Heated tumor tissue is imitated by lower acetone concentrations, since the tissue permittivity decreases with increasing temperature [22,23]. In this study, we use concentrations between 30 vol% and 50 vol% acetone. The compositions and dielectric properties of the MUTs used in our experiments are presented in Figure 4 and Table 1. It must be emphasized that we are aware that the resulting permittivity change is much larger than the change that can be expected as a result of hyperthermia. The aim of the current investigations is development of reliable quantitative estimation approaches based on UWB imaging, initially without the challenge of very a low signal-to-noise-ratio.

The first experiment is dedicated to the detection of slightly changing permittivity imitating temperature increase in the tumor during hyperthermia. The measurement starts with the reference material (MUT_ref_) with a minimum concentration of acetone (30 vol%). To achieve a slight change of dielectric properties, 1 mL of the respective MUT solution (12 mL) is gradually substituted for a 1 mL mixture with concentration 55 vol% acetone and 45 vol% distilled water until after 19 repetitions, the concentration of the acetone in the mixture will be equal to 50 vol%. At each step, the signals of 128 channels will be recorded. 

The second experiment is dedicated to the imaging and quantification of the dielectric properties change in the tumor region. To obtain a high image reconstruction quality, the antenna array with installed antennas rotates to 24 positions with an angle step of 15 degrees, so that 3072 signals are received for each measurement, instead of 128 in the first type of measurements. Five MUTs are under investigation—the dielectric properties of MUT_ref_ are defined and the dielectric properties of MUT (*T* = 1.4) are estimated based on the presented methodology and compared with the measured values by means of dielectric spectroscopy.

## 3. Results

### 3.1. Qualitative Investigation

In this section, we present the results of our investigation of the detection of dielectric properties change due to increasing temperatures via UWB technology. 

**Figure 5:**
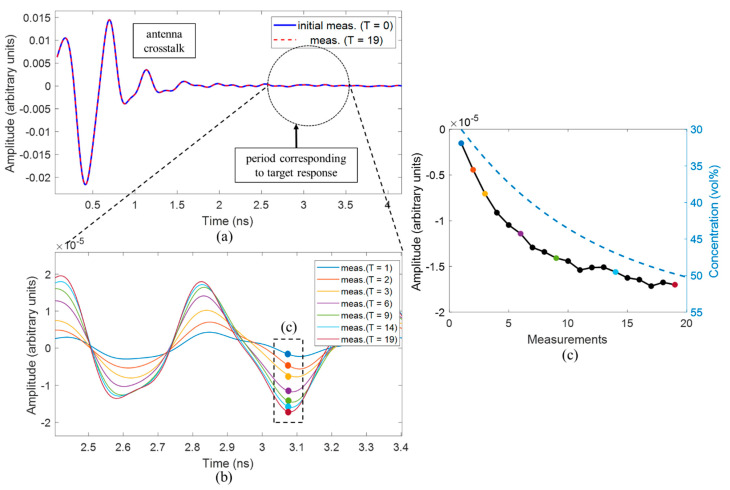
(**a**) Raw signals of channel 1, (**b**) corresponding differential signals, (**c**) correlation between amplitude of the differential signal and changing concentrations of MUT (dashed line).

The measured raw signals of an exemplary channel are presented at Figure 5a. Obviously, the static clutter (antenna crosstalk) is much stronger than the target response, but even the small change of the dielectric properties of the target can be detected. Figure 5b shows the differential signals $s_{n}(t,T>0)-s_{n}(t,T=0)$. The correlation between decreasing relative permittivity of the target and the difference amplitude in the period representing the target response is presented in Figure 5c. The trend of this curve corresponds to the expected behavior—the magnitude of the differential signal in this range confirms the change in acetone concentration, where the successive steps decrease with the ongoing procedure. The important and promising aspect of this result is the almost monotony of the magnitude of the differential signal. It shows the capability of the radar system and the measurement setup and exhibits that even small permittivity variations are detected.

**Figure 6:**
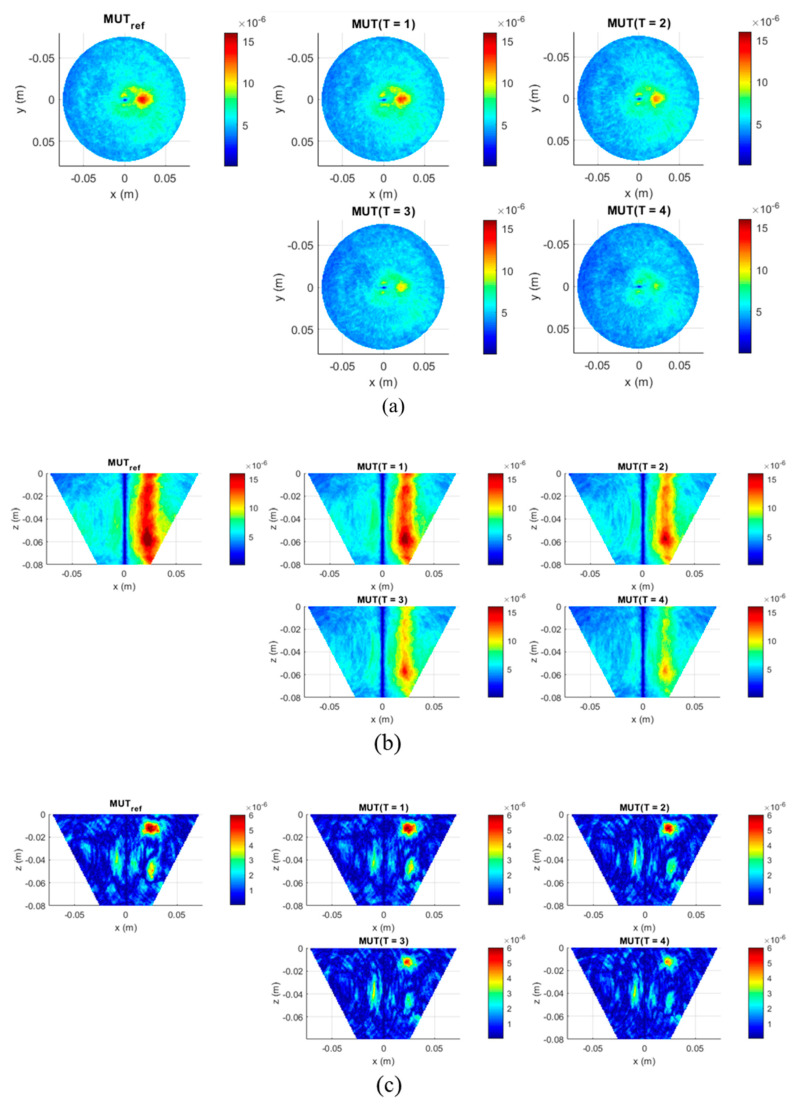
UWB images of the five phantoms demonstrating the correlation between intensity of the image $I(r_{0},T)$ reconstructed via Equation (6) and the decreasing permittivity of the tumor imitate in (**a**) xy-slice, depth z = −2 cm and (**b**) in xz-slice, y = 0. The magnitude of the common DAS imaging (Equation (4)) is shown in (**c**). Color bar represents the intensity of the image in the arbitrary units.

At the preprocessing stage of our second measurement scenario before imaging of the measured non-differential signals, we need to exclude the influence of the plastic tube on the results. For this purpose, we use a measurement of only background material and a measurement with background material plus a tube filled with background material. Their difference estimates the effect caused by the presence of the plastic tube in the phantom and then is subtracted from all five measurements with tumor imitate. After this, 3D images are generated from these cleaned data via the adapted DAS algorithm (Equation (6)). The imaging domain has dimensions as defined in the description of Figure 3, with a resolution step of 1 mm for all dimensions. The images of measurements with MUT_ref_ and MUT (*T* = 1…4) are presented in Figure 6. The minimum in the center of the phantom is caused by antennas crosstalk removal by means of rotational average value subtraction of each channel [34].

As we can see from Figure 6a,b, we are able not only to localize the tumor correctly, but also see the qualitative correlation between the decreasing intensity in the tumor area and the decreasing relative permittivity of the tumor. The comparison of the images in Figure 6b,c shows the clear advantage of the imaging approach via the adapted DAS algorithm (Equation (6)) against the basic version (Equation (4)). These results show that the UWB measurement system is able to qualitatively detect the induced relative permittivity changes and accordingly the temperature changes are spatially distributed in the tumorous tissue.

### 3.2. Quantitative Estimation

**Figure 7:**
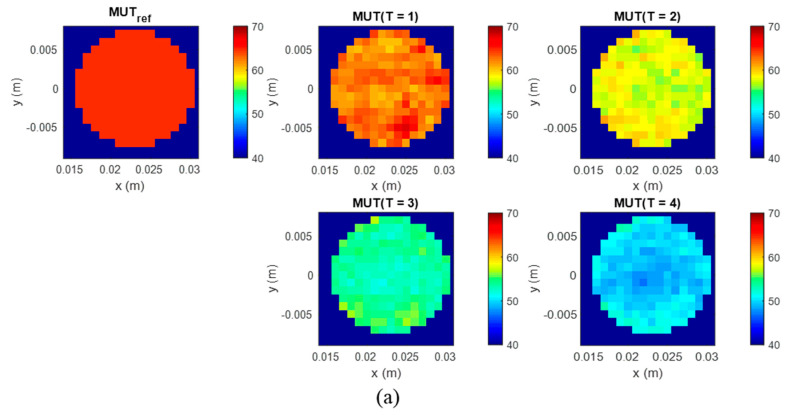

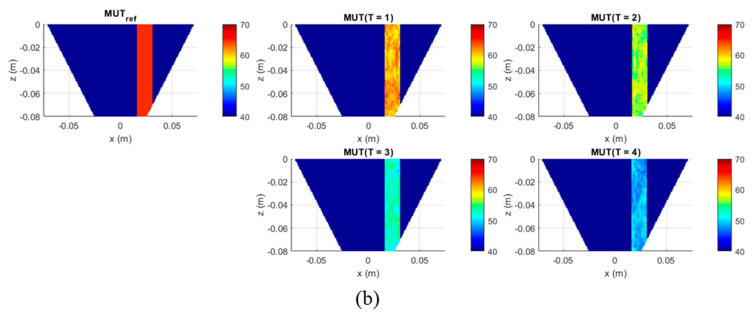
Distribution of the estimated relative permittivity ${\underline{\hat{\varepsilon }}}_{t}(r_{0},\omega ,T)$ at 2 GHz based on Equation (10) in the tumor area (**a**) in xy-slice, depth z = −2 cm and (**b**) in xz-slice, y = 0. Color bar represents the relative permittivity value.

We can estimate the dielectric properties based on the images reconstructed via the adapted DAS algorithm (Figure 6). The image values represent the data at the whole frequency band of the UWB measurement setup. Relative permittivity is estimated for each of four phantom’s images $I(r_{0},T=1\ldots 4)$ by means of Equations (9) and (10), where the frequency dependent dielectric properties of the reference scenario are known. The distribution of the estimated relative permittivity exemplarily at 2 GHz in the tumor region of MUT (*T* = 1…4) is presented in Figure 7.

**Figure 8:**
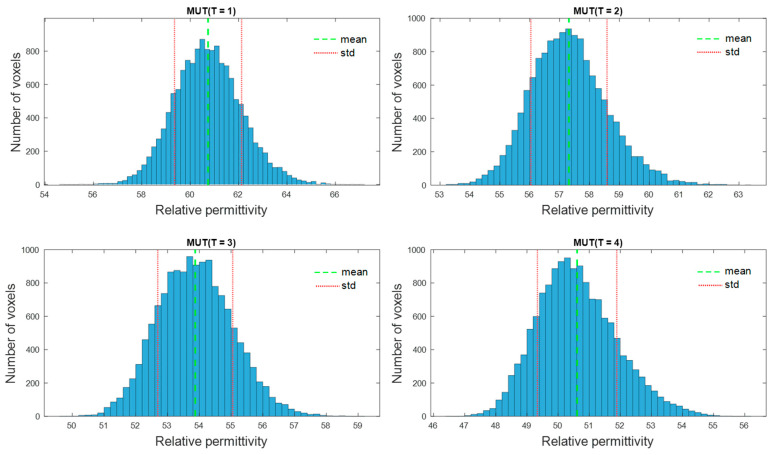
Distribution of the estimated relative permittivity values at 2 GHz in the 3D tumor area. Mean value is shown in green and standard deviation in red.

The numerical distribution of the values of estimated relative permittivity in the 3D imaging domain is presented in Figure 8. The data show a nearly symmetric distribution.

**Figure 9:**
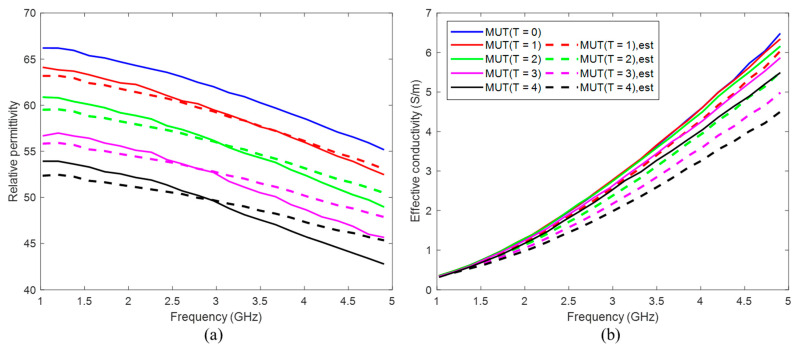
Estimated mean relative permittivity (**a**) and effective conductivity (**b**) in the 3D tumor area as function of frequency (dashed lines) and measured by means of dielectric spectroscopy (solid lines).

According to Equation (10), the permittivity estimation algorithm is applied from 1 to 5 GHz. The effective conductivity is calculated via Equation (1). The comparison of the mean values of the estimated dielectric properties of MUT (*T* = 1…4) in the 3D tumor region and measured ones (Figure 4) is shown in Figure 9.

## 4. Discussion

In this contribution, we have investigated the detectability of dielectric properties change mimicking the temperature increase by means of M-sequence UWB technology. The experimental results show that investigated permittivity variations can be detected and qualitatively imaged. The tumor representing image magnitude decreases with decreasing dielectric contrast between tumor and background material. Although the imaged tumor permittivity variations are still larger than those expected during hyperthermia treatment, the received signals of the first experiment point out that smaller variations can also be adequately imaged. 

Furthermore, we suggested an approach for translating this image information into quantitative values of estimated dielectric properties in the tumor region. The estimation algorithm is based on the temperature dependent changes of the reflection coefficient at the boundary between background material and tumor mimicking tissue. According to Equation (9), this approach simplifying assumes that the behavior of the ongoing changing complex reflection coefficients can be linearly described by the ratio of the real-valued UWB image values. Therefore, according to Equation (10), it also assumes that this relationship is frequency independent and valid for the real part as well as for the imaginary part of the permittivity. Obviously, due to different dispersity of tissues and tissue mimicking materials (see Figure 4), this assumption does not hold in reality. Therefore, even if the estimated curves tend to follow the true curves more or less well, frequency dependent deviations between estimated and true relative permittivity values arise (Figure 9a). These deviations decrease with decreasing dielectric variations, which is advantageous for hyperthermia monitoring because due to the low temperature increase between 4 and 8 °C, only very low permittivity decreases will occur. In addition, it has to be emphasized that the application of hyperthermia monitoring does not aim to reconstruct the permittivity curve over a wide range of frequency, but it does aim to estimate the temperature robustly. So, due to the fact that UWB sensing involves wide frequency band information, it will practically be sufficient to estimate the permittivity within an optimal frequency band. In case of the experiments done in this work, Figure 9a points out that the frequency range between 2.8 GHz and 3.5 GHz allows the most robust estimation. Additionally, Figure 9b reveals that the relative deviations between estimated and measured effective conductivity are significantly higher than between the corresponding relative permittivity curves and that they increase with increasing frequency. Hence, the real part of the estimated permittivity seems to be more suitable for temperature monitoring than the imaginary part. 

In continuation of our previously published work [25,29], this paper presents initial investigations towards quantitative non-invasive tissue temperature estimation based on UWB measurements. The current findings have to be proven by means of further measurements, influencing parameters have to be identified and still existing limitations have to be overcome. During thermal treatment, undesired hot spots can appear in surrounding organs which should not be heated and even a small increase of temperature in such organs (e.g., spinal cord) can be dangerous for the patient. Thus, it is also important to estimate the temperature in the non-target area. In further investigations, the suggested estimation algorithm has to be extended to be able to estimate the temperature in the tumor region, as well as in the surrounding tissues. The type of surrounding tissue has a significant influence on the resulting possibilities. Low-water content tissue (e.g., tumor surrounding fat tissue) provides a good contrast to tumorous tissue which improves the visibility of the tumor in the image, but it is mostly temperature independent [14], so it will be very challenging to detect temperature changes inside of fat tissue based on microwave sensing. In cases when tumorous tissue is surrounded by muscle or glandular tissues whose dielectric properties can be almost similar, the challenge arises from the low dielectric contrast between the tissues, and therefore, from low reflectivity in the tumor region. 

In future works, the temperature monitoring system is planned to be inserted in a microwave hyperthermia setup in order to get closer to a real clinical scenario. A more complex phantom structure with several different tissue mimicking materials needs to be used in these experiments.

## Figures and Tables

**Table 1:** Materials under test used in the experiments.

Symbol	Composition	*ε* at 2 GHz
MUT_ref_	70 vol% ddH2O + 30 vol% acetone	64.5–i11.6
MUT (*T* = 1)	65 vol% ddH2O + 35 vol% acetone	62.3–i11.7
MUT (*T* = 2)	60 vol% ddH2O + 40 vol% acetone	58.8–i11.6
MUT (*T* = 3)	55 vol% ddH2O + 45 vol% acetone	55.5–i11.2
MUT (*T* = 4)	50 vol% ddH2O + 50 vol% acetone	52.3–i10.6
MUT_bg_	100 vol% cream	33.2–i5.4
